# Painful ejaculation in a patient with pelvic venous insufficiency

**DOI:** 10.1016/j.jvscit.2022.07.014

**Published:** 2022-08-07

**Authors:** Sebastian Cifuentes, Jorge H. Ulloa, Emelyn Van Uden, Valentin Figueroa, Antonio Solano, Ana C. Montenegro

**Affiliations:** a Vascular Surgery Department, Fundacion Santa Fe de Bogota University Hospital, Bogota, Colombia; b Universidad de Los Andes Medical School, Bogota, Colombia; c Internal Medicine Department, Fundacion Santa Fe de Bogota University Hospital, Bogota, Colombia

**Keywords:** Gonadal vein, Painful ejaculation, Pelvic angiography, Pelvic varicosities, Pelvic venous insufficiency, Venous embolization

## Abstract

Pelvic venous insufficiency (PVI) is common in women but has been rarely diagnosed in men. The clinical manifestations include varicocele and pelvic disturbances; however, we were unable to find a previous description of painful ejaculation as a symptom of PVI. We present the case of a 36-year-old man with a 7-year history of severe sharp ejaculatory pain. PVI was suspected after previous treatment attempts. The diagnosis was confirmed by descending phlebography, and he underwent coil embolization of the pelvic vessels and phlebotonic therapy. At 6 months after treatment, he reported a 75% improvement in his condition. Therefore, painful ejaculation should be considered an uncommon manifestation of PVI.

Pelvic venous insufficiency (PVI) was first described around the 1850s but was studied in depth in the 1950s, when it was mostly correlated with female pelvic congestion syndrome (PCS).^1^^,^^2^ It is a common pathology in women, with a clinical presentation that can include hemorrhoids and leg, vulvar, and pudendal varicosities.^3^ In men, it has been correlated with varicocele formation and pelvic disturbances. In the present report, we have described an uncommon manifestation of PCS: ejaculatory pain. Usually, patients will have attended several specialists before an accurate diagnosis will be reached and the proper treatment started. Although PVI has been reported in the vascular surgery literature, it has not been previously correlated with ejaculatory alterations.^4^ The singularity of our case and the multidisciplinary management between vascular surgery and urology have contributed significantly to a better understanding and workup of male patients with PVI. The patient provided written informed consent for the report of his case details and imaging studies.

## Case report

A 36-year-old man had attended the urology department with a 7-year history of severe sharp pressure-like pelvic pain associated with tenesmus and congestion during and after ejaculation and also after prolonged sitting, affecting his job as a pilot. The patient rated his pain as 10 of 10 on the visual analog scale (VAS). His medical history included dyslipidemia, a 3-mm left kidney stone, and left varicocele that had been surgically treated with ligation at the inguinal canal during adolescence. The patient had been previously assessed by several specialties, including urology, internal medicine, and psychiatry, and had repeatedly received analgesic medication and antibiotics for a presumptive diagnosis of prostatitis.

On arrival at our institution, the urology department had conducted the initial assessment and ruled out prostatitis, a common cause of ejaculatory pain, via pelvic ultrasound and fluid culture. The physical examination did not evidence abnormalities. However, given the unclear etiology, medical history, and atypical clinical manifestation, PVI was suspected, and the patient was referred to the vascular surgery department.

**Fig 1:**
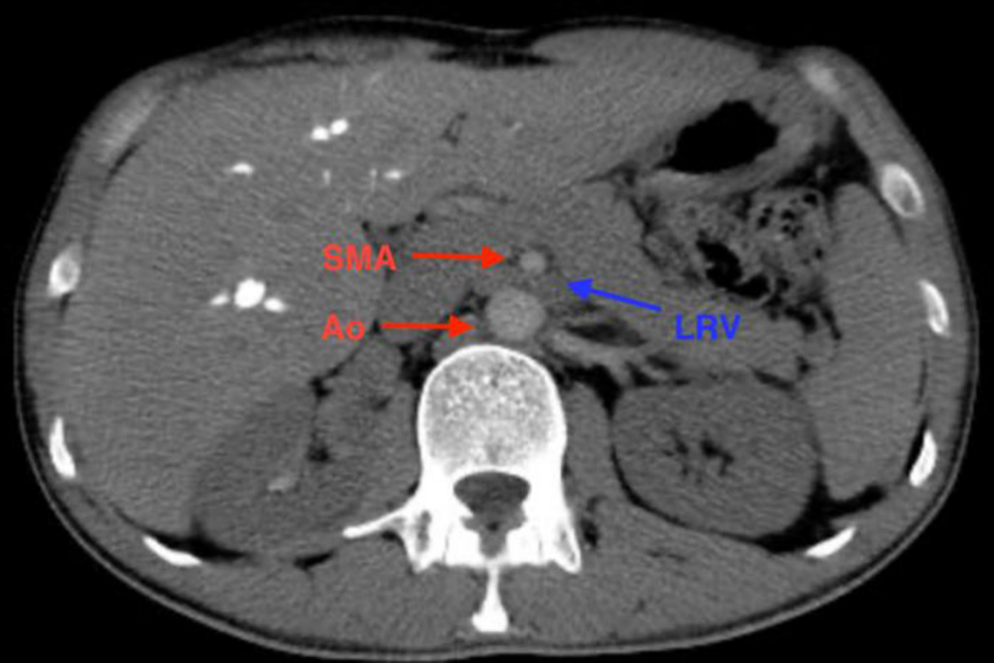
Axial view of pelvic computed tomography scan demonstrating an uncompressed left renal vein, ruling out nutcracker syndrome. *Ao,* Aorta; *LRV,* left renal vein; *SMA,* superior mesenteric artery.

**Fig 2:**
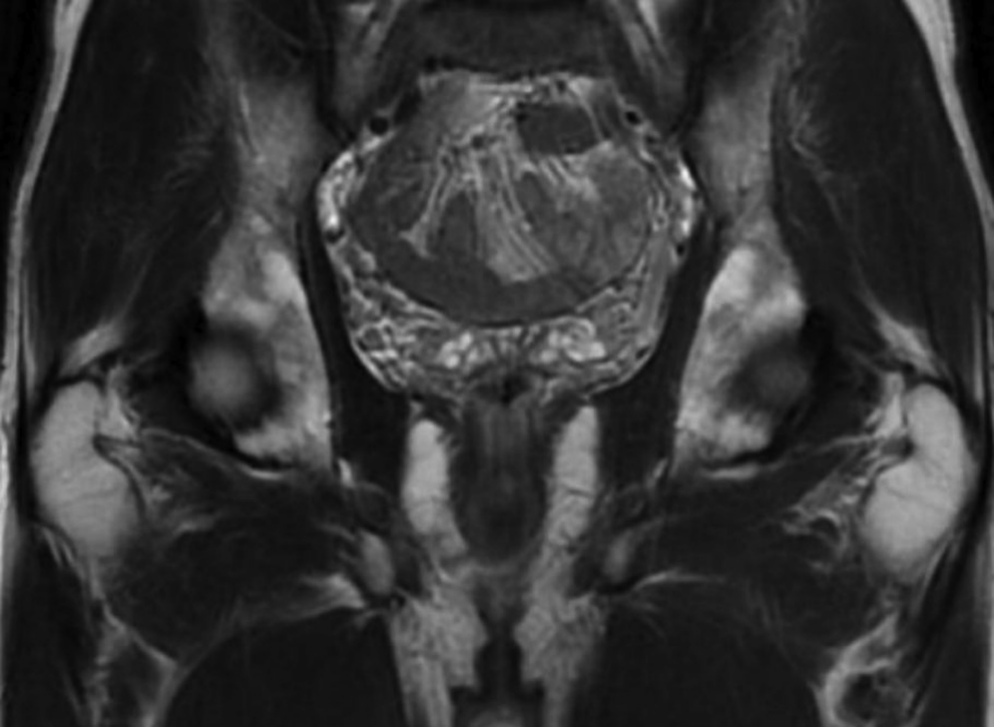
Coronal view of pelvic magnetic resonance image showing enlarged bilateral periprostatic venous plexus ≤8 mm in diameter.

**Fig 3:**
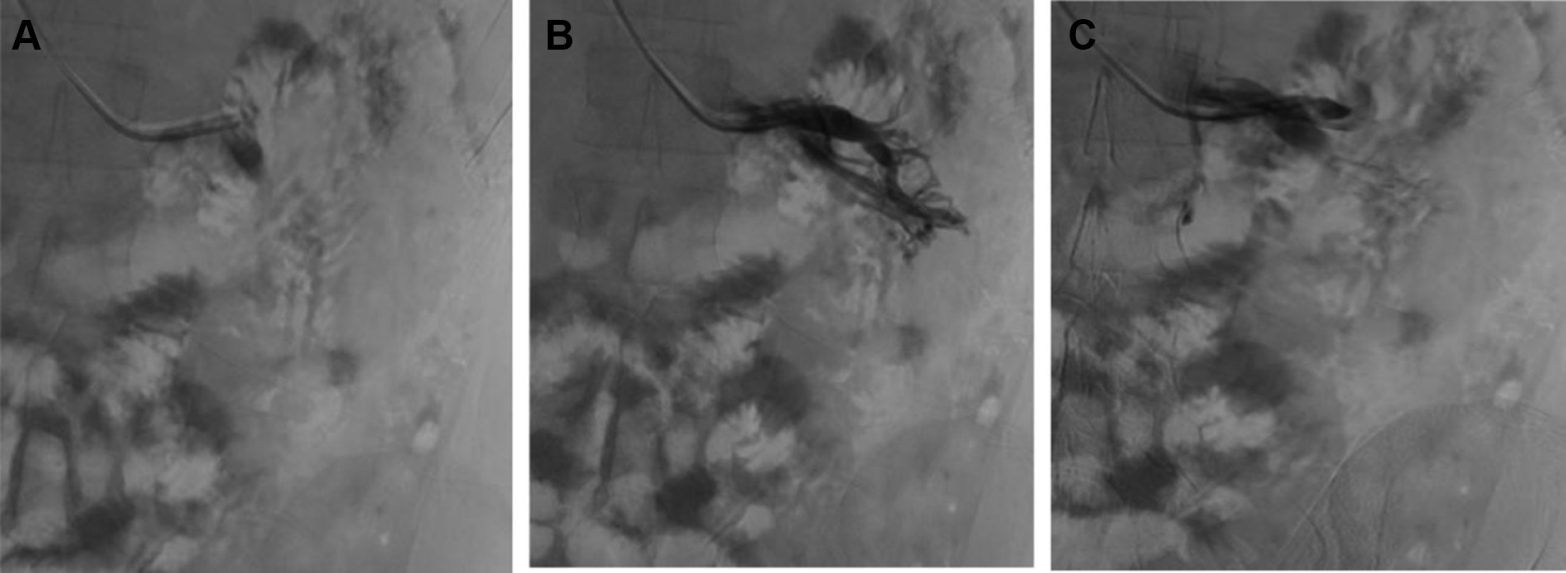
**A-C,** Descending venography injection sequence showing dilated plexus and left gonadal vein during a Valsalva maneuver. Contrast reflux compatible with venous insufficiency and pelvic congestion syndrome (PCS) can be seen. Presacral opacification was present, evidence of periprostatic venous dilation. The unobstructed contrast flow through the left renal vein also ruled out the presence of nutcracker syndrome.

The initial evaluation included lower limb venous duplex ultrasound, with normal findings. Next, pelvic computed tomography was performed, which ruled out nutcracker syndrome (Fig 1). In addition, pelvic magnetic resonance imaging showed prominent bilateral periprostatic venous dilations ≤8 mm in diameter (Fig 2). The iliocaval axis was patent bilaterally, without signs of thrombosis. Intravascular ultrasound was not available. The diagnosis of PCS was confirmed through a therapeutic left selective descending venography with manual injection. It evidenced significant venous reflux, dilated plexuses, and moderate dilation (6 mm) of the left gonadal vein with contrast reflux with and without the Valsalva maneuver. The right gonadal vein was visualized, and no signs of disease were found. Additionally, periprostatic and presacral venous plexus opacification was observed crossing the midline (Fig 3).

**Fig 4:**
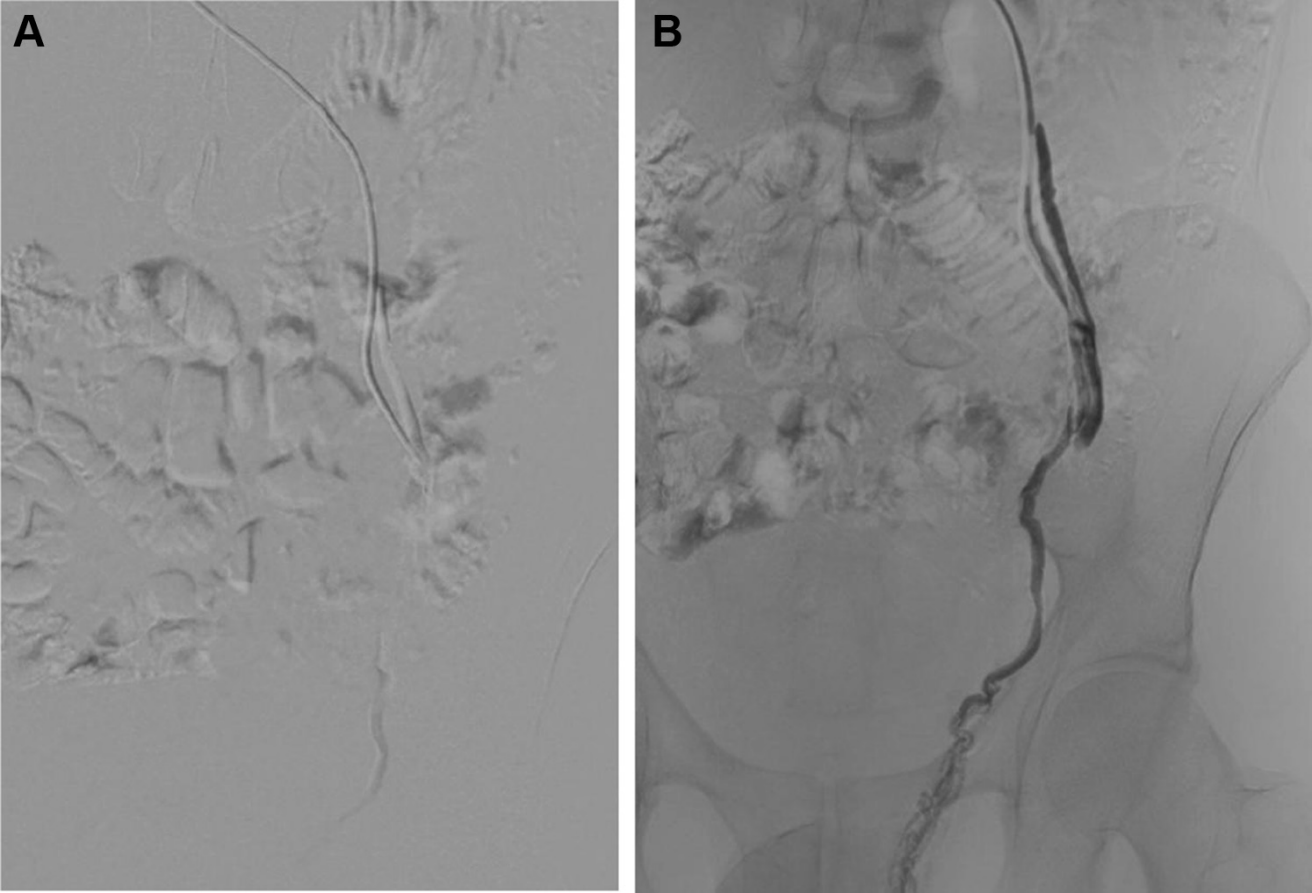
**A** and **B,** Descending venography showing introduction of the Progreat 0.025-in. microcatheter and positioning in the confluence of the pampiniform plexus and a gonadal vein tributary. The pampiniform plexus coalesced proximal to the testis to form the gonadal vein.

**Fig 5:**
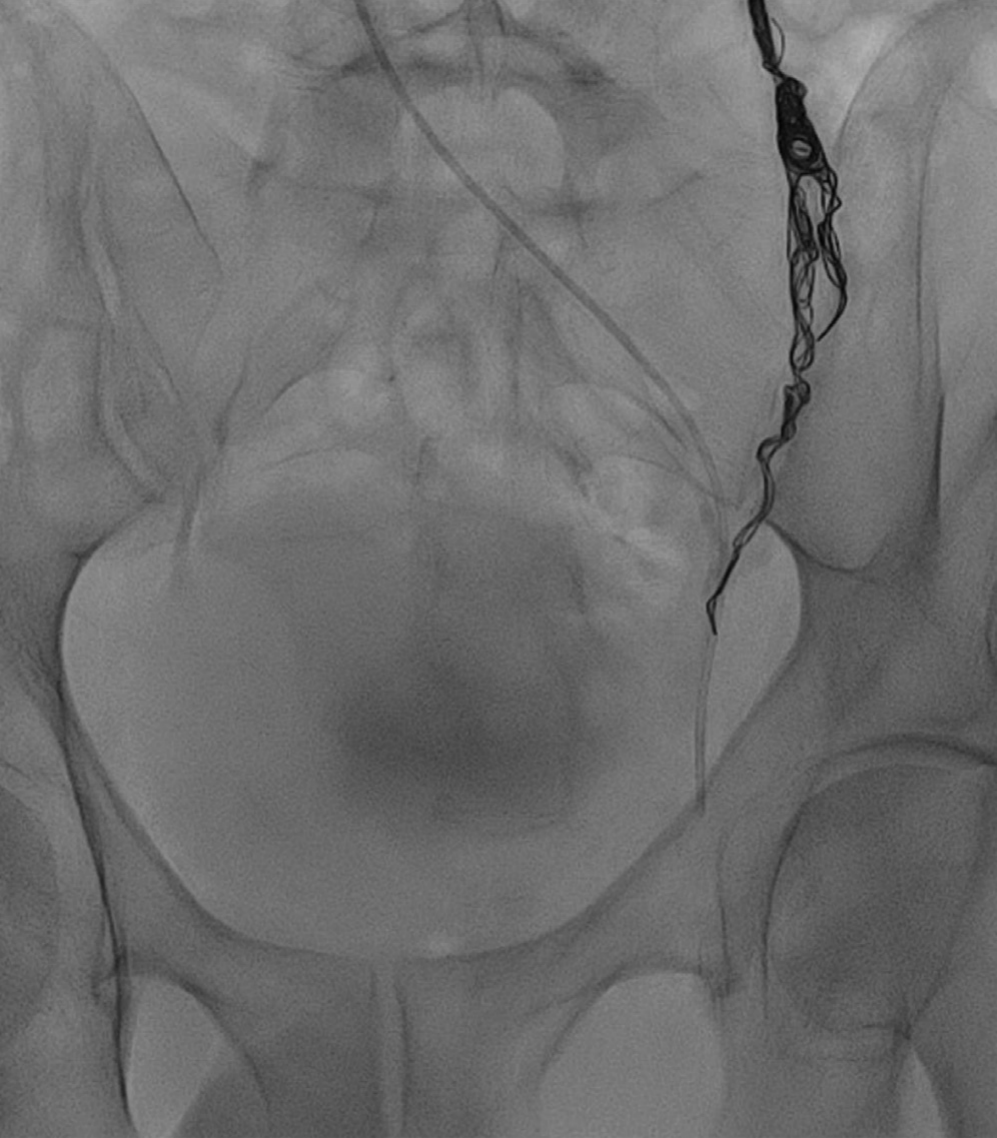
Descending venography after embolization showing adequate embolization of the gonadal vein.

During the same surgical session, a Progreat 0.025-in. microcatheter (Terumo Interventional Systems, Tokyo, Japan) was inserted up to the confluence of the pampiniform plexus and a gonadal vein tributary (Fig 4). The gonadal vein was successfully embolized using two 8-mm × 30-cm Penumbra 400 coils (Penumbra Inc, Alameda, CA; Fig 5). In addition, no compression or varicosities were identified in the internal iliac and femoral veins. The procedure was well-tolerated, and no adverse events occurred. A phlebotonic agent (MPFF [micronized purified flavonoid fraction], 1000 mg, once daily) was prescribed as adjuvant therapy. The patient experienced rapid clinical improvement. After 1 week, the reported VAS pain level had decreased from 10 to 7. One month later, he had reported a 50% improvement in pain and was able to complete intercourse. After 6 months of phlebotonic therapy, his clinical improvement had reached 75%, with mild pain (3 points on the VAS) during and after ejaculation.

## Discussion

PVI refers to the pathophysiologic mechanism of retrograde flow through incompetent pelvic veins, usually resulting in PCS. Its correlation with pelvic pain in women was established in the 1950s,^1^^,^^2^ and the first case of PVI in men was reported years later. However, it is an underdiagnosed disease with an unknown prevalence.^5^ PVI in men will usually debuts with varicocele and in women with dull pelvic pain.^1^^,^^3^ Orgasm-associated pain in men has a reported prevalence of 1% to 9.7%.^6^ Nonetheless, it is a poorly understood phenomenon by most specialties. Some described causes have been seminal vesicle alteration, sexual neurasthenia, sexually transmitted diseases, antidepressant use, inflammation of the prostate, ejaculatory duct obstruction, and after radical prostatectomy.^7^^,^^8^ However, none of these were identified in our patient. A few reports have described arteriovenous malformation as a possible cause of ejaculatory pain.^4^ However, we could not find any literature correlating PVI or PCS with this type of pain.

Under normal conditions, the lower extremity veins will flow into the external iliac vein (EIV), and the pelvic veins (eg, obturator, gluteal, internal pudendal veins) will flow into the internal iliac vein (IIV). The junction of the EIV and IIV results in the common iliac vein.^9^ Very few valves are present in the EIV, IIV, and common iliac vein. Therefore, the most common cause of PVI is valvular incompetence of the gonadal vein near its junction with the renal vein.^10^ Although primary valvular incompetence is the most common etiology of PVI in men and women, it can be worsened during pregnancy owing to the prolonged increased intra-abdominal pressure, leading to chronic venous distension and valve malfunction. Compression of normal anatomic structures can result in PVI, such as the nutcracker and May-Thurner syndromes. Our patient had probably had primary incompetence, because no risk factors for secondary damage were identified.

Pelvic pain is common in PCS; however, the pathophysiology has not been clearly established. The potential mechanisms of pain include the mechanical mass effect of the varicosities on the adjacent nerves^11^ and a molecular etiology with the release of vasoactive peptides (eg, substance P, neurokinins A, B, endothelins, calcitonin gene-related peptide) from the enlarged intima,^12^ contributing to inflammation and subsequent pain receptor activation.^11^ Symptoms such as pressure, heaviness, dysuria, and dyspareunia will frequently be exacerbated by sexual intercourse.^9^^,^^13^ In our patient, prostate contraction during ejaculation could have led the enlarged veins to exert pressure on the periprostatic nerves. The present case portrays an uncommon situation for vascular surgeons, which should increase awareness of the occurrence of PVI in men. Because this disease in men is usually characterized by varicocele,^9^^,^^14^ surgical correction can only treat the local peritesticular incompetence, without addressing the concomitant incompetent pelvic venous valves.^15^^,^^16^

Regarding the diagnosis, one study evaluated the usefulness of transrectal ultrasound for patients with prostatodynia.^5^ However, transrectal ultrasound showed that 87% of these men had had PVI.^5^ Although more invasive, venography is also indicated to confirm the diagnosis of PVI and can be used as a diagnostic and therapeutic tool at a single intervention.^17^ Coil embolization can be considered when an incompetent vessel has been identified, with confirmatory descending venography during the same surgical session. Additionally, 1000 mg of MPFF once daily has demonstrated benefits for treating PVI, significantly improving the clinical severity scores and quality of life and reducing thrombosis rates.^18^^,^^19^ We observed favorable clinical outcomes with the implemented medical and surgical treatment.

## Conclusions

Finally, with the present case, we have described a rare manifestation of PVI, which should increase awareness about how challenging this disease can be to diagnose in men. Our patient had required a complex diagnostic workup with assessment by multiple specialties. Uncommon pelvic pain in men requires abdominal and pelvic venous Doppler ultrasound and venous phase pelvic computed tomography because of the possibility of PVI.^20^ Our report has contributed to the scarce literature regarding PVI in men. PVI can significantly affect patients’ quality of life, with a delay in diagnosis and treatment owing to underrecognition by physicians. Therefore, vascular surgeons must have a high clinical suspicion index when evaluating patients with atypical clinical presentations.
